# The effects of social situation cues and negative smoking outcome expectancies on attentional bias among smokers

**DOI:** 10.3389/fpsyt.2025.1264539

**Published:** 2025-08-25

**Authors:** Danling Lin, Yumeng Fan, Jia Wang, Lin Chen, Haide Chen

**Affiliations:** ^1^ College of Education, Zhejiang Normal University, Jinhua, China; ^2^ Zhejiang College of Security Technology, Wenzhou, China; ^3^ School of Psychology, Zhejiang Normal University, Jinhua, China

**Keywords:** attentional bias, smokers, social situation cues, smoking-related cues, outcome expectancy

## Abstract

**Introduction:**

Although numerous findings support the triggering effect of drug-related cues on drug-seeking behavior among addicts, there is a paucity of studies investigating whether attentional bias toward these cues can be moderated by social factors. The present study aimed to examine the influence of social situation cues and negative smoking outcome expectancies on attentional bias among smokers.

**Methods:**

In study 1, 36 smokers and 34 nonsmokers completed a modified dot-probe task that incorporated social situation cues as priming stimuli. In study 2 (N = 58), a sentence construction task was introduced to further explore how negative smoking outcome expectancies affect attentional bias influenced by social situation cues.

**Results:**

Study 1 found that attentional bias toward smoking-related cues was more pronounced in the smoking social situation cue condition than in the non-smoking social situation cue condition. Study 2 further found that when negative smoking outcome expectancies were activated, attentional bias toward smoking-related cues might be reduced in the smoking social situation cue condition.

**Discussion:**

These results indicated that attentional bias could be sharpened not only by social situation cues but also by negative smoking outcome expectancies. This study provides preliminary evidence concerning the potential flexibility of attentional biases toward drug-related cues among individuals facing addiction issues.

## Introduction

Tobacco use remains one of the leading preventable causes of premature death globally, accounting for over 8 million fatalities each year due to its association with respiratory and cardiovascular diseases (1). Although early cessation significantly reduces mortality and morbidity (2), relapse rates continue to be alarmingly high, underscoring the need for a deeper understanding of the cognitive mechanisms contributing to persistent smoking behavior.

A pivotal factor in relapse is attentional bias—the tendency for smoking-related cues to disproportionately capture and retain attention (3). Empirical studies consistently demonstrate that an increased attentional bias toward smoking-related stimuli predicts relapse in smoking behavior (4–6). For instance, utilizing a modified emotional Stroop task, Waters et al. (4) found that individual differences in attentional bias could predict subsequent smoking during cessation attempts.

Several theoretical frameworks have sought to elucidate how attentional bias influences substance use behavior. The Incentive-Sensitization Theory (7) provides a crucial framework for understanding the development of attentional bias. According to this theory, repeated parings of smoking-related cues with nicotine’s rewarding effects lead to the sensitization of motivational pathways, rendering these cues highly salient and capable of automatically capturing attention (8). One study examined this theory, and the findings were generally aligned with the behavioral predictions derived from it (9). Furthermore, the Goal Theory of Current Concerns (10) posits that an individual’s pursuit of a specific goal initiates a latent, time-binding cognitive process—referred to as a current concern—that prompts emotional responses and enhances notice, recall, think about and act on cues associated with the goal pursuit.

However, much research has concentrated on isolated smoking-related item cues (e.g., cigarette package; 11), often overlooking the significance of situational contexts. Social situations are strongly correlated with smoking behavior (12), as smokers frequently perceive shared cigarette use as a means of fostering social connections (13). Smokers are more liked to smoke in social settings (e.g., restaurants) than in restricted environments (e.g., workplaces; 14). In a non-smoking environment, smokers may refrain from smoking due to conformity or obedience (15). Moreover, situational cues alone—even in the absence of direct smoking stimuli—can elicit cravings (16), potentially exacerbating attentional bias (17, 18). Despite this evidence, it remains unclear whether and how social situation cues influence attentional bias.

While Incentive-Sensitization Theory and the Goal Theory of Current Concerns elucidate the automatic capture of attention by smoking-related cues, the dual-process model posits that reflective processes—such as negative outcome expectancies (e.g., anticipated social stigma)—may modulate these effects. Complementing the aforementioned theories, the dual-process model (19) suggests that smoking behavior results from an interaction between impulsive processes (e.g., automatic attentional bias toward cues) and reflective processes (e.g., outcome expectancies). Smoking outcome expectancies are the subjective perception and expectation of smokers regarding the consequences of their smoking behavior (20). Negative outcome expectancies are associated with quit attempts (21, 22), whereas positive expectancies reinforce substance use initiation (23). Consequently, attentional bias may be more pronounced when impulsive processes prevail (e.g., in the presence of highly salient cues) and weaker when reflective processes counteract them (e.g., when negative outcome expectancies are prominent).

Given these theoretical perspectives, the present study aims to investigate how social situation cues influence attentional bias and whether negative outcome expectancies moderate this relationship. We hypothesize that: (1) non-smoking social situation cues will reduce attentional bias towards smoking-related cues; (2)negative outcome expectancies will reduce attentional bias in smoking social situations by engaging reflective processes.

## Study 1

In Study 1, both smokers and nonsmokers were recruited online as participants to investigate the impact of social situation cues on attentional bias through a modified dot-probe task. It was hypothesized that smokers would demonstrate higher attentional bias scores in the smoking social situation cue condition compared to the non-smoking social situation cue condition. In contrast, nonsmokers were not expected to exhibit significant differences in attentional bias scores between the two cue conditions.

## Methods

### Design

A 2×2 factorial design was used, with group (smoker *vs*. nonsmoker) serving as the between-subjects factor and condition (smoking social situation cues *vs*. non-smoking social situation cues) as the within-subjects factor. The dependent variable was attentional bias, operationalized as the difference in reaction times between inconsistent and consistent trials (RT _inconsistent_ - RT _consistent_; 24) when participants responded to smoking-related item cues. This research protocol was reviewed and approved by the Institutional Review Board (IRB) of the institute of Psychological and Brain Sciences at Zhejiang Normal University.

### Participants

Current daily smokers were defined as individuals who had smoked every day during the past month (25). The sample comprised 36 participants in the current daily smoker group and 34 in the nonsmoker group, with average ages of 20.53 and 21.44 years, respectively. All participants were male and were required to exclude any history of organic brain diseases or psychiatric disorders, as well as any substance addiction other than tobacco. There were no significant differences in demographic variables between the two groups. Smoking-related characteristics of smokers are presented in **Table 1**.

**Table 1:** Smoking-related characteristics of smokers.

Characteristics	Smoker (n = 36)	nonsmoker (n = 34)
	*M* ± *SD*	*M* ± *SD*
Age of first cigarette	16.44 ± 2.13	–
Age of starting smoking	18.03 ± 2.16	–
Number of daily cigarettes	10.29 ± 6.00	–
Nicotine dependence	0.45 ± 0.32	–
Smoking urges	3.36 ± 1.32	–

### Measurements

#### Demographic questionnaire

According to a study of young smokers (25), the demographic questionnaire used in our study was included standard demographic items (e.g., age, gender, education) and smoking-related items (e.g., the number of cigarettes consumed per day).

#### Fagerström test for nicotine dependence scale

Nicotine dependence was assessed using the Fagerström Test for Nicotine Dependence Scale (26) which consists of 6 items (e.g., “*How soon after you wake up do you smoke your first cigarette*?”). Higher scores indicate a greater degree of nicotine dependence.

#### Questionnaire on smoking urges-brief

Smoking urges were measured using the Questionnaire on Smoking Urges-Brief (27), comprising 10 items (e.g., “*I have a desire for a cigarette right now*.”). Responses were recorded on a 7-point Likert scale ranging from 1 = strongly disagree to 7 = strongly agree). Higher scores reflect an increased intensity of smoking urges.

### Materials

**Figure 1:**
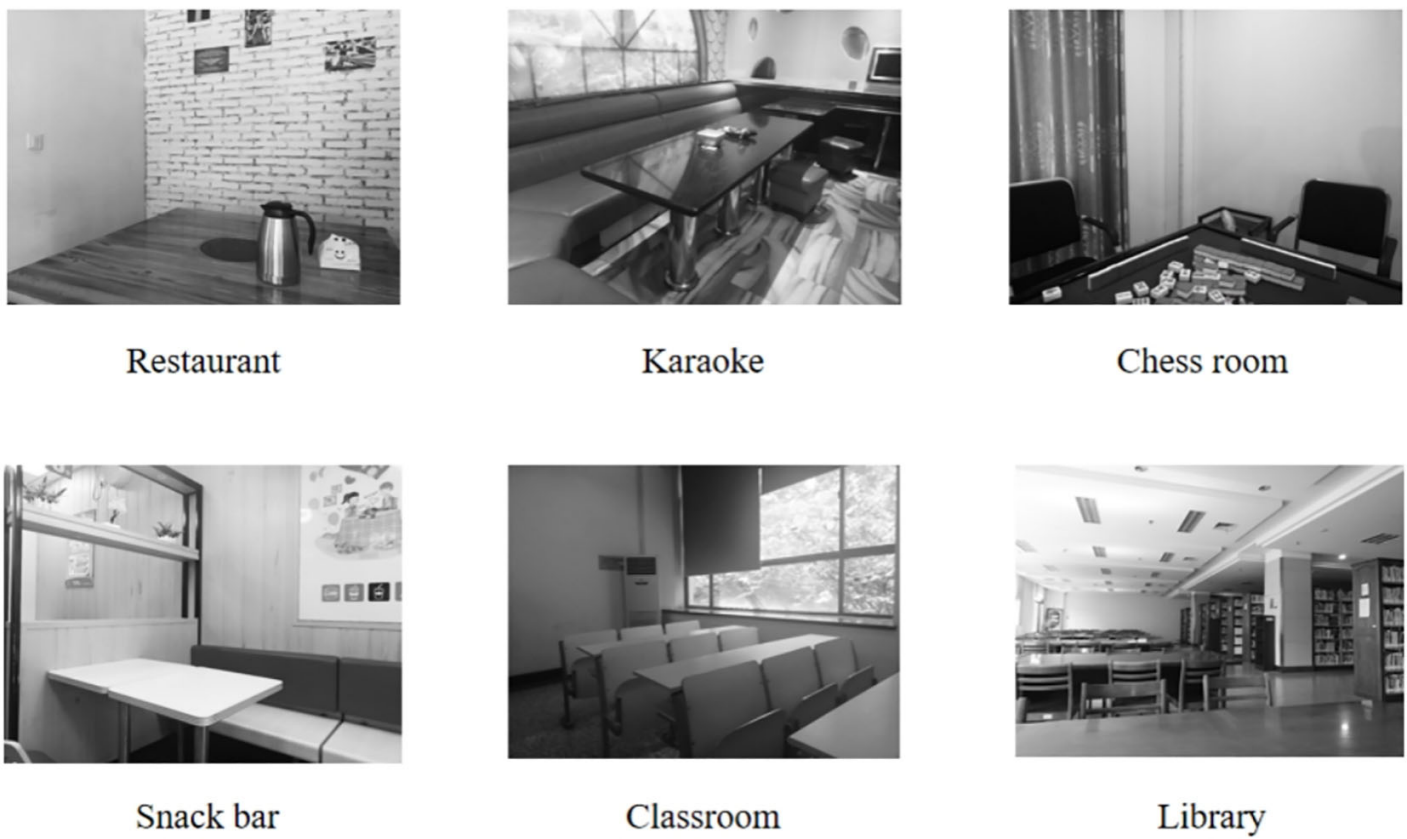
Smoking social situation cues vs. non-smoking social situation cues.

**Figure 2:**
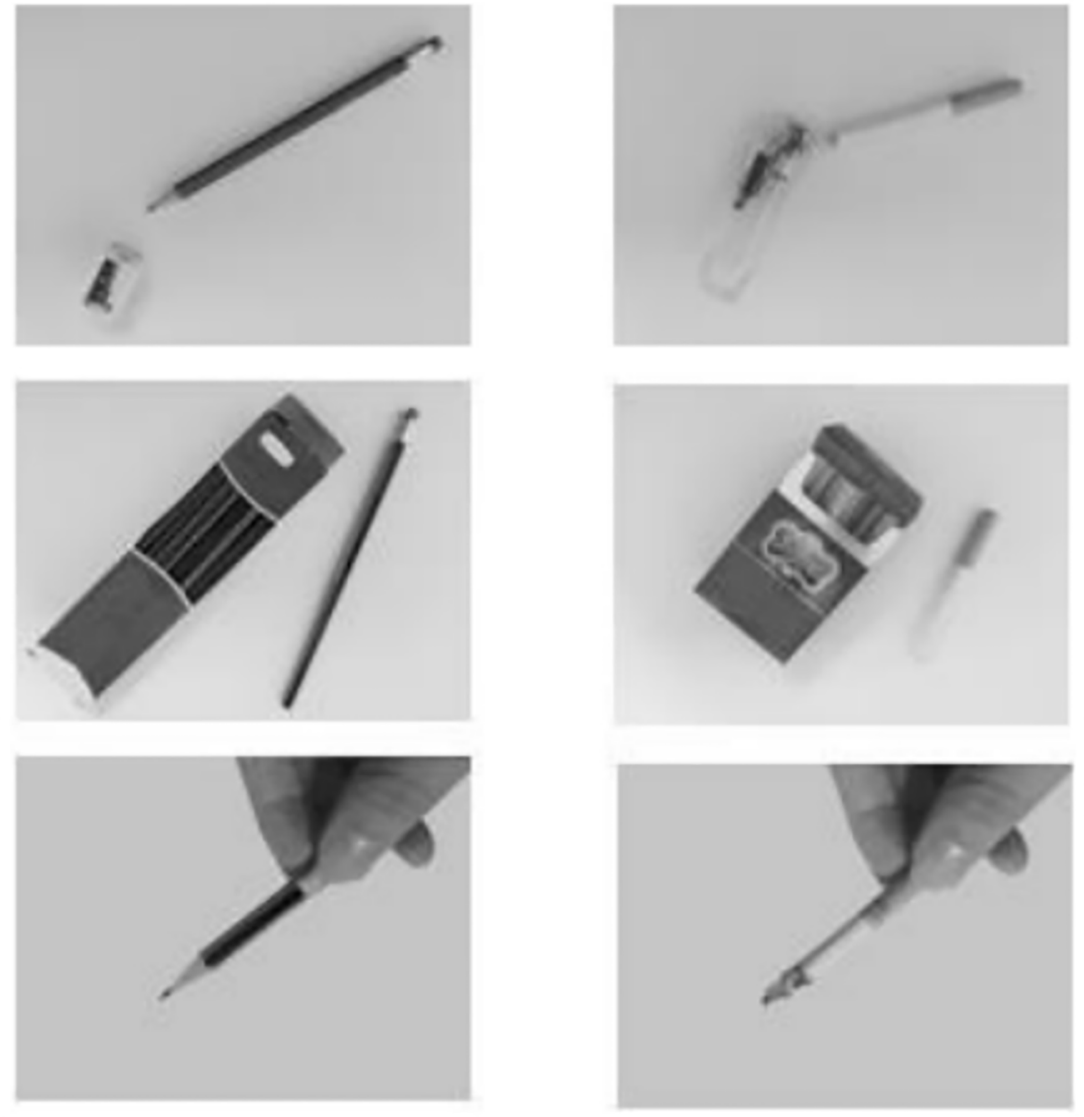
Neural item cues vs. smoking-related item cues.

We administered a modified dot-probe task that featured either images depicting smoking social situations (e.g., karaoke) or non-smoking social situations (e.g., library). These contextual images (see **Figure 1**) were displayed prior to each trial and were selected based on evidence that such situations are the most common smoking/nonsmoking places (28). Within each trial, paired images consisting of one smoking-related item (e.g., cigarette) and one neural item (e.g., pencil) were presented simultaneously (see **Figure 2**). Smoking-related/neural items were chosen from standardized sets used in prior research (29). All images were evaluated in terms of valence and arousal during a pilot study (*N* = 55) and only images with >80% agreement in thematic classification were included. Ultimately, we selected those images that matched for overall composition and perceptual characteristics (e.g., brightness and visual complexity).

### Procedure

**Figure 3:**
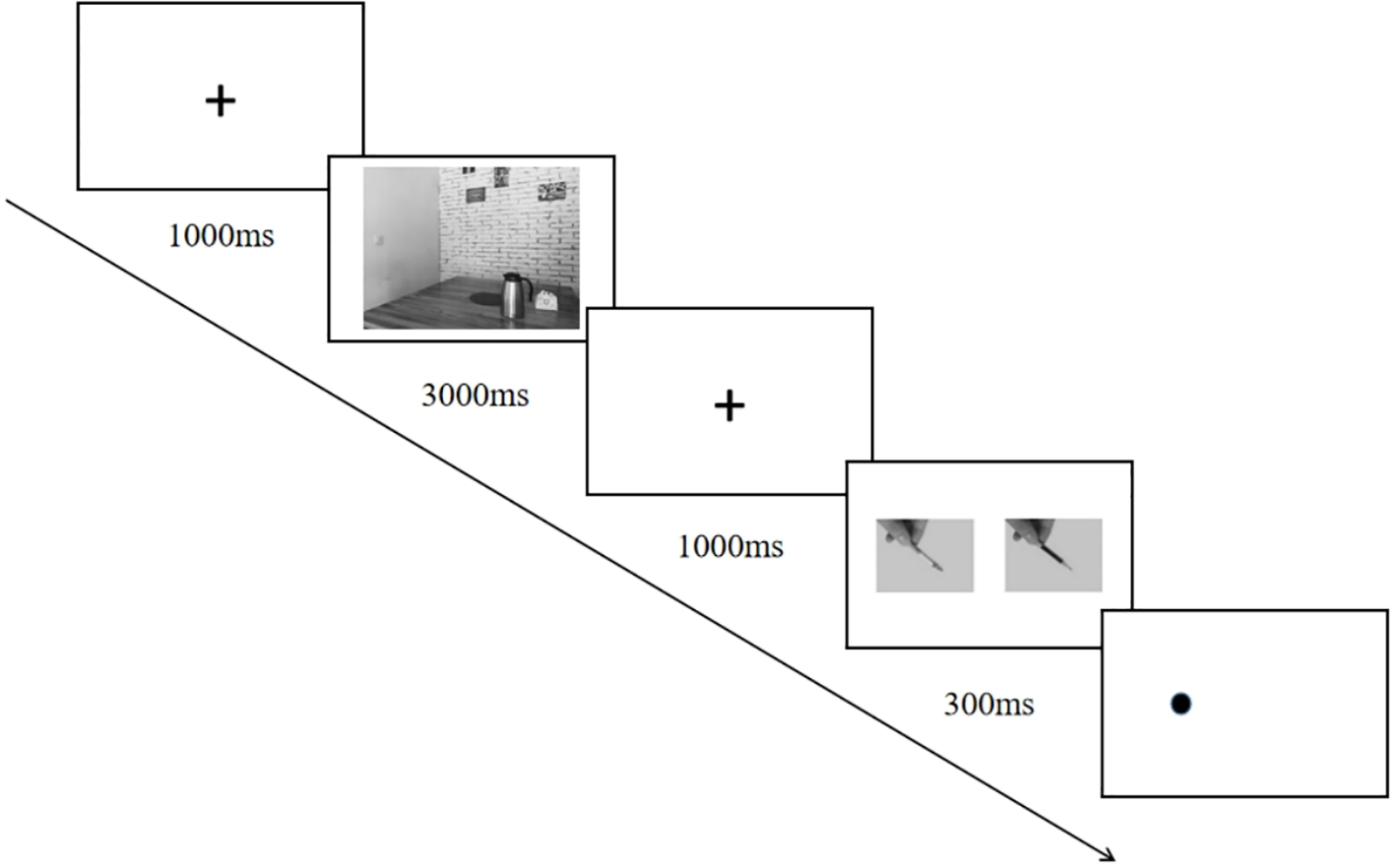
The modified dot-probe task.

Participants provided informed consent before completing the self-report measures. Subsequently, the strength of attentional bias was assessed using a modified version of the dot-probe task (see **Figure 3**). Prior to the formal trial session, participants were instructed to complete six practice trials of the modified dot-probe task. Each trial commenced with a fixation point displayed at the center of the screen for 1000 ms. This fixation point was then replaced by an image depicting a social situation, which remained on-screen for 3000 ms. Following this, the fixation point reappeared for another 1000 ms before being replaced by a pair of pictures presented for 300 ms. The picture pair consisted of one smoking-related item and one neutral item, with their positions (left or right relative to center) fully counterbalanced across trials. A probe point subsequently appeared either on the left or right side of the screen. Standard instructions required participants to press the “F” key in response to probes appearing on the left and to press the “J” key in response to probes appearing on the right as quickly and accurately as possible. If the position of the probe matched that of the preceding smoking-related item picture, it constituted a consistent condition; otherwise, it was classified as an inconsistent condition. The probability distribution between consistent and inconsistent conditions within each block was set at 50% each. Participants completed six practice trials to familiarize themselves with this experimental task. The formal experiment comprised one block consisting of 36 trials designed to ensure continuous exposure to social situation cues; thus, its duration was controlled within 8 minutes.

## Results

### Reaction time and attentional bias scores among smokers and nonsmokers under two different social situation cue conditions

The descriptive statistics of reaction time and attentional bias scores of smokers and nonsmokers across two social situation cue conditions are presented in **Table 2**. Notably, the attentional bias scores of smokers were positive in the smoking social situation cue condition, indicating that smokers exhibited an attentional bias toward smoking-related item cues within this context.

**Table 2:** Reaction time (ms) and attentional bias scores (*M* ± *SD*) of smokers and nonsmokers in two social situation cue conditions.

Condition	Consistency	Smoker (n *=*36)	Nonsmoker (n *=*34)
Smoking social situation cues	Consistent	401.63 ± 47.72	381.67 ± 53.63
	Inconsistent	411.57 ± 63.07	370.17 ± 43.72
	Attentional bias scores	9.93 ± 27.65	-11.50 ± 25.05
Non-smoking social situation cues	Consistent	412.64 ± 54.81	376.87 ± 42.26
	Inconsistent	407.54 ± 57.77	373.99 ± 43.16
	Attentional bias scores	-5.11 ± 34.03	-2.88 ± 27.51

### Differences in attentional bias scores between smokers and nonsmokers across two social situation cue conditions

A repeated measures ANOVA was conducted to assess the differences in attentional bias scores between smokers and nonsmokers under the two social situation cue conditions. The interaction between condition and group was found to be significant, *F*(1, 68) = 5.93, *p* = 0.018, partial *η* ^2^ = 0.080. However, the main effect of condition was not significant, while the main effect of group was marginally significant, *F*(1, 68) = 3.86, *p* = 0.054, partial *η* ^2^ = 0.054.

**Figure 4:**
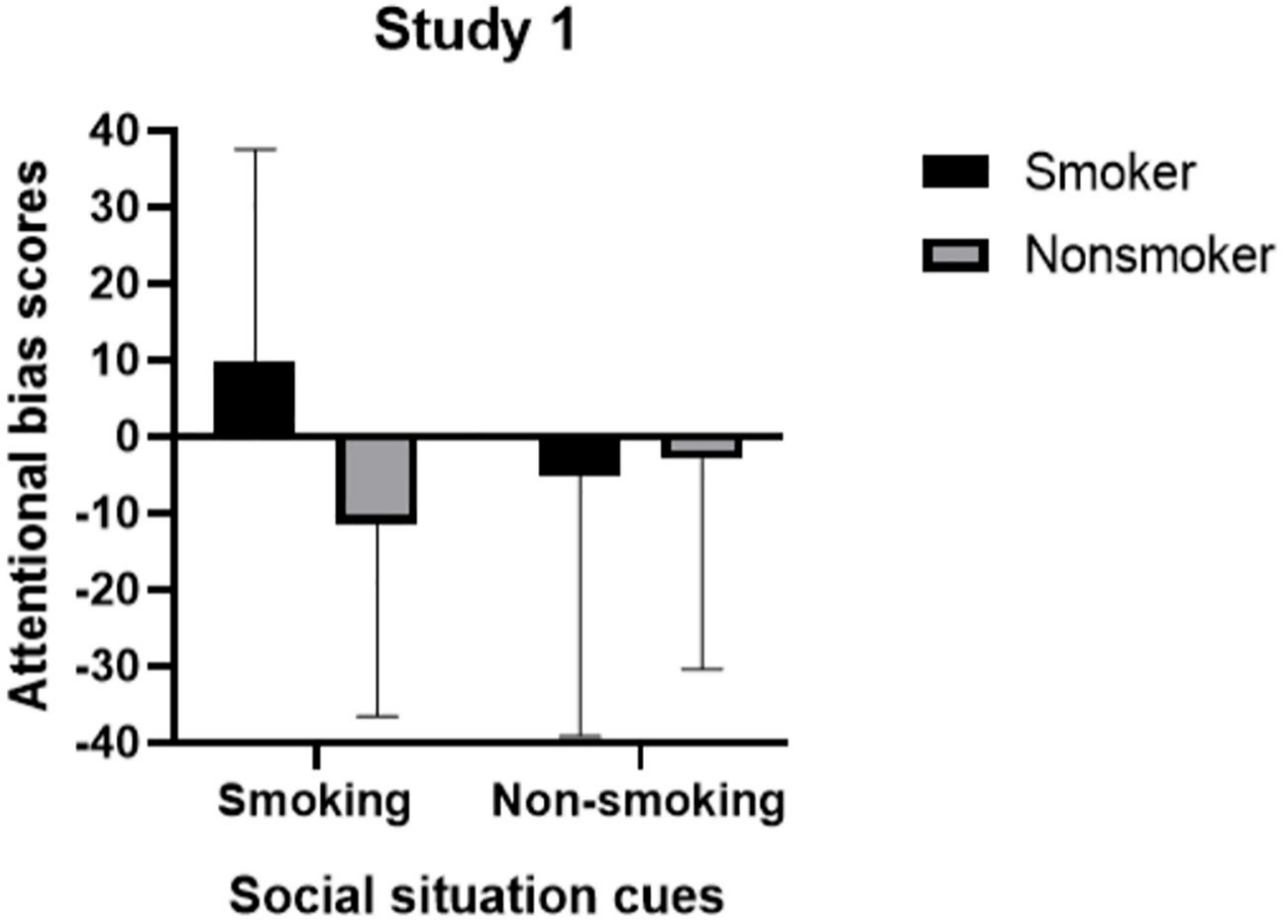
Attentional bias scores of smokers and nonsmokers in smoking and non-smoking social situation cue conditions.

Subsequent simple effects analyses were performed to further investigate the differences in attentional bias scores between the two groups across different types of social situation cue conditions. The results revealed a significant difference in attentional bias scores between smokers and nonsmokers specifically within the smoking social situation cue condition, *F*(1, 68) = 11.51, *p* = 0.001. Notably, smokers demonstrated significantly higher attentional bias scores compared to nonsmokers. However, no significant difference was observed in attentional bias scores between these groups during the non-smoking social situation cue condition, *F*(1, 68) = 0.09, *p* = 0.765 (see **Figure 4**).

## Discussion

In study 1, we explored how social situation cues influence attentional bias. These findings suggest that non-smoking social situation cues may reduce attentional bias toward smoking-related item cues. As anticipated, non-smokers displayed no evidence of an attentional bias for smoking-related item cues regardless of which social situation cue condition they were exposed to. For smokers, however, their attentional bias was positive in the smoking social situation cue condition and was significantly greater than those recorded during exposure to non-smoking social situation cues. And the negative attentional bias scores observed in the non-smoking social situation cue condition suggest that smokers did not exhibit an attentional bias toward smoking-related item cues. This result is consistent with previous research findings. Situations previous associated with positive smoking experiences can independently elicit strong subjective cravings among smokers (16). Furthermore, smokers are more likely to engage in smoking behavior in smoking social situations (e.g., restaurants), whereas such behavior is less common in non-smoking social situations (e.g., workplaces; 14). Non-smoking social situations may reduce attentional bias toward smoking-related stimuli, thereby limiting the likelihood of smoking behavior.

## Study 2

Smoking behavior is influenced not only by external social situations but also by internal cognitive processes. Therefore, in study 2, we incorporated a sentence construction task (30) to further investigate the impact of negative smoking outcome expectancies on the attentional bias as affected by social situation cues. We hypothesized that in the smoking social situation cue condition, the attentional bias scores for smoking-related item cues would be lower in the experimental group (which activated negative smoking outcome expectancies) compared to the control group. However, in the non-smoking social situation cue condition, we anticipated no significant difference between the two groups.

## Methods

### Design

A 2×2 factorial design was used, with group (experimental group *vs*. control group) serving as the between-subjects factor and condition (smoking social situation cues *vs*. non-smoking social situation cues) as the within-subjects factor. The dependent variable was attentional bias, operationalized as the difference in reaction times between inconsistent and consistent trials (RT _inconsistent_ - RT _consistent_) when participants responded to smoking-related item cues. This research protocol was reviewed and approved by the Institutional Review Board (IRB) of the institute of Psychological and Brain Sciences at Zhejiang Normal University.

### Participants

Sixty current daily smokers (all male) were recruited online using identical selection criteria as those applied in study 1. These participants were randomly assigned to either the experimental or control group. However, two individuals did not complete all aspects of the experiment as required. Consequently, the final sample comprised 28 participants in the control group and 30 participants in the experimental group. Smoking-related characteristics of these smokers are detailed in **Table 3**.

**Table 3:** Smoking-related characteristics.

Characteristics	Experimental group (n =30) *M* ± *SD*	Control group (n = 28) *M* ± *SD*	*t*
Age of first cigarette	15.47 ± 1.85	15.89 ± 2.32	0.78
Age of starting smoking	17.20 ± 1.44	17.21 ± 1.73	0.04
Number of daily cigarettes	7.87 ± 4.63	9.18 ± 4.95	1.04
Nicotine dependence	6.87 ± 1.10	7.54 ± 0.88	2.55 *
Smoking urges	3.25 ± 1.48	3.77 ± 1.43	1.36

*, *p* < 0.05.

### Measurements

Same as study 1.

### Materials

The images used in the modified dot-probe task were consistent with those utilized in Study 1. In terms of materials for the sentence construction task, text items pertained to dimensions associated with negative smoking outcome expectancies from the Smoking Consequences Questionnaire (SCQ; 31). A total of three text materials were presented sequentially. A total of three text materials were presented sequentially, one of which read as follows.

*On the weekend, I was enjoying a karaoke session with a group of friends. While engaging in conversation with those seated next to me, I felt the urge to smoke a cigarette. However, upon observing my friends and the surrounding environment, I decided against it. I reflected that if I were to smoke at that moment, it might make others feel _________. Additionally, I was concerned about __________.*

### Procedure

Participants provided informed consent prior to completing self-report measures and a baseline test assessing attentional bias through a classical dot-probe task (32). Next, participants were instructed to complete six practice trials of the modified dot-probe task. Prior to the formal trial session, the experimenter conducted a manipulation aimed at including negative smoking outcome expectancies within the experimental group. Participants in this group received additional materials designed to activate these negative smoking outcome expectancies. Subsequently, they were asked to imagine or recall a social situation in which they could not smoke based on the initial text materials and to articulate their reasons for abstaining from smoking in that context. In contrast, participants in the control group did not undergo any manipulation. Upon completion of the modified dot-probe task, participants of the experimental group responded to a question intended to assess the effectiveness of the negative smoking outcome expectancies manipulation: “*What do you think is the likelihood that you will smoke in smoking social situations?”* (1 = not at all, 5 = very likely). A chronological summary of Experiment 2 can be found in **Table 4**.

**Table 4:** Chronological summary of experiment 2 procedures.

Procedure	Time	Measures
Baseline assessments of attentional bias	Day 1	The classical dot-probe task
Negative outcome expectancies intervention	Day 2	Sentence construction task
Test on attentional bias in different socal situation cue conditions	Day 2	Modified dot-probe task (same as experiment 1)
Manipulation check	Day 2	An evaluation question

The procedure on the second day consists of a total of 3 stages, and there is no time gap between the three stages.

## Results

### Baseline scores for attentional bias between the experimental and control groups

An independent t-test was used to examine whether there was any difference in the baseline scores of attentional bias toward smoking-related item cues between both groups. The result indicated no significant difference in the baseline attentional bias scores between participants in the experimental and control groups (see **Table 5**), *t*(56) = 0.90, *p* = 0.371.

**Table 5:** Reaction time (ms) and baseline scores of attentional bias (*M* ± *SD*) in the experimental and control group.

Conditions	Experimental group (n = 30) *M* ± *SD*	Control group (n = 28) *M* ± *SD*	*t*
Consistent	399.10 ± 38.86	408.29 ± 36.82	0.92
Inconsistent	404.13 ± 38.29	416.29 ± 39.47	1.19
Baseline scores of attentional bias	5.03 ± 13.18	8.00 ± 11.79	0.90

### Negative smoking outcome expectancies manipulation check

To evaluate whether our manipulation was effective, we conducted another independent t-test focusing on negative smoking outcome expectancies. The analysis revealed that participants in the control group more strongly endorsed intention to smoke in smoking social situations (*M* = 4.75) compared with those in the experimental group (*M* = 3.97), *t*(56) = 2.23, *p* = 0.030. Conversely, individuals in the experimental group perceived themselves as less likely to engage in smoking under similar circumstances than their counterparts in the control condition. Therefore, it can be concluded that our manipulation was successful among participants assigned to the experimental group.

### Attentional bias scores of the experimental and control groups in two social situation cue conditions

The descriptive statistics of attentional bias scores are presented in **Table 6**. The attentional bias scores of the experimental group were lower than those for the control group in both the smoking social situation cue condition and the non-smoking social situation cue condition. In the experimental group, participants did not exhibit any attentional bias toward smoking-related item cues in either condition. Conversely, participants in the control group demonstrated an attentional bias toward smoking-related item cues specifically in the smoking social situation cue condition. Consistent with findings from Study 1, no attentional bias toward smoking-related item cues was observed in the non-smoking social situation cue condition.

**Table 6:** Attentional bias scores (*M* ± *SD*) of the experimental and control group in two social situation cue conditions.

Condition	Experimental group (n = 30)	Control group (n = 28)
	*M* ± *SD*	*M* ± *SD*
Smoking social situation cues	-9.70 ± 13.85	8.64 ± 6.08
Non-smoking social situation cues	-6.37 ± 18.74	-4.21 ± 14.81

### Differences in attentional bias scores between experimental and control groups across two social situation cue conditions

A repeated measures ANOVA was conducted to assess differences in attentional bias scores between the experimental and control groups across various types of social situation cue conditions while controlling for factors such as age, occupation, education level, and nicotine dependence. The interaction between condition and group was found to be significant, *F*(1, 50) = 6.71, *p* = 0.013, partial *η* ^2^ = 0.118, as was the main effect of group, *F*(1, 50) = 11.78, *p* = 0.001, partial *η* ^2^ = 0.191.

**Figure 5:**
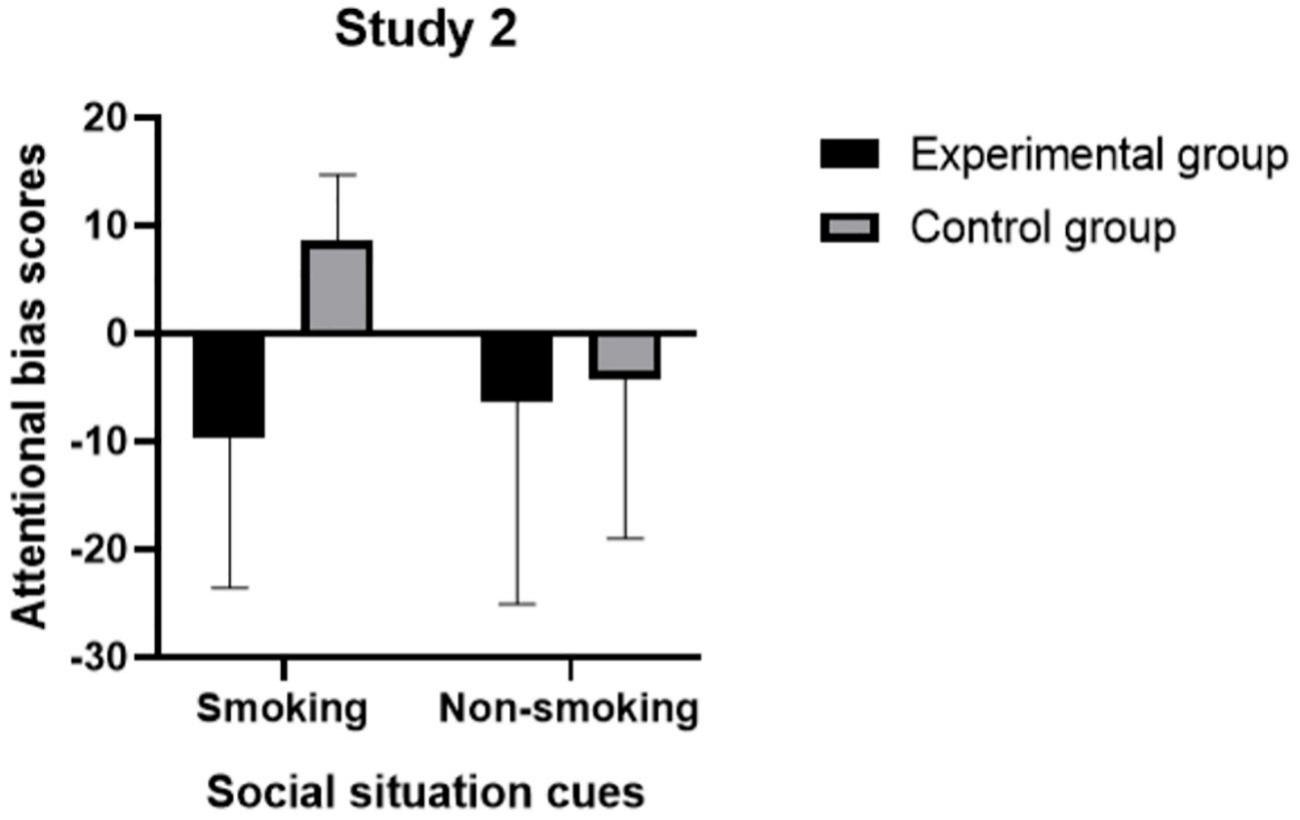
Attentional bias group of two groups in smoking and non-smoking social situation cue conditions.

Subsequent simple effects analyses were used to further investigate differences in attentional bias scores between the two groups under different types of social situation cue conditions. The results indicated that there were significant differences in attentional bias scores between groups in the smoking social situation cue condition, *F*(1, 50) = 31.23, *p* = 0.000; specifically, attentional bias scores were significantly greater among participants in the control group compared to those in the experimental group. However, no significant difference was noted between groups regarding attentional bias scores in the non-smoking social situation cue condition, *F*(1, 50) = 0.54, *p* = 0.468 (see **Figure 5**).

## Discussion

In Study 2, we investigated whether negative smoking outcome expectancies influenced attentional bias under smoking social situation cue condition. The experimental design of Study 2 demonstrated that negative smoking outcome expectancies played a reflective role in modulating attentional bias toward smoking-related item cues. Smokers whose cognitive states were primed with negative smoking outcome expectancies did not exhibit significant attentional bias toward smoking-related item cues, even when exposed to smoking social situation cue s. These findings suggest that attentional bias may be reduced not only by external environment as well as by internal cognitive processing mechanisms.

## General discussion

Attentional bias in smokers is dynamically modulated by both environmental cues and internal cognitive processes. The present findings indicate that attentional bias toward smoking-related item cues can be reduced by both non-smoking social situation cues and the activation of negative smoking outcome expectancies.

The effect of non-smoking social situation cues might be explained as follows: smokers are more likely to engage in smoking behaviors in social situations (12). Past experiences of positive smoking behavior in specific situations may elicit strong subjective cravings, even in the absence of direct smoking-related cues such as cigarette package (16). However, in non-smoking social situations, smokers are unable to engage in smoking behaviors, which may result in reduced craving levels and potentially reduce attentional bias toward smoking related item cues. According to cue-response theory (33), exposure to smoking social situation cues can trigger a range of behavioral and cognitive responses to smoking-related item cues. In contrast, due to the lack of reinforcement in non-smoking social situations, these situations may not develop strong associations with smoking behavior, thereby reducing the attentional bias.

The attentional bias observed in smoking social situations supports the premise of the dual-process model that chronic smoking reinforces automatic associations (8). Social situations may disproportionately affect the impulsive system, thereby creating an imbalance that perpetuates addictive behaviors. In contrast, removing the attentional bias of smokers in smoking social situations through negative outcome expectancies demonstrates the ability of the reflective system to regulate attention. These results align with and extend the dual-process model (19), emphasize the preemptive role of reflective processes. Priming negative outcome expectancies reduced attentional bias even in smoking social situations, suggesting top-down cognitive regulation may override automatic cue reactivity.

This study provides insights into the mechanisms underlying the reduction of attentional bias, offering both theoretical and practical implications. Theoretically, it supports the dual-process model by confirming the interplay between impulsive and reflective processes. It also expands existing theories of attentional bias, such as Incentive-Sensitization Theory, by emphasizing the combined influence of environmental and cognitive factors beyond the traditional focus on the rewarding properties of addictive substances. In practice, both non-smoking social situations and the activation of negative smoking outcome expectancies demonstrate potential for smoking cessation interventions. First, reducing opportunities for smoking in social situations is crucial. Negative attentional bias scores in non-smoking social situations (e.g., library) suggest these environments may actively reduce smoking-related attentional capture. Smoke-free social situations may gradually weaken the association between social situations and smoking behavior, thereby reducing cravings and facilitating smoking cessation. Second, leveraging the reflective function of negative smoking outcome expectancies can aid in quitting efforts. Our findings suggest that activating these expectancies enhances self-control and reduces attentional bias in social situations. During smoking cessation programs, promoting awareness of the adverse consequences of smoking and helping individuals internalize stable negative smoking outcome expectancies could be effective strategies. For instance, integrating anti-smoking messages into social norms may reinforce these cognitive shifts.

This study presents several limitations that warrant consideration in future research. Firstly, the utilization of static social situation images (see **Figure 1**) as priming cues and the controlled laboratory environment may restrict ecological validity. To more accurately reflect real-world experiences, subsequent studies could implement more immersive methodologies, such as ecological momentary assessment (EMA) or virtual reality (VR) environments, which facilitate dynamic and naturalistic social interactions. Secondly, our participant recruitment was conducted online, which did not employ a random sampling method. Additionally, the all-male sample may limit generalizability to female populations. Future research should adopt random sampling methods and include diverse gender samples to enhance external validity. Thirdly, given that real-life smoking outcome expectancies can be significantly influenced by environmental factors, further investigation is needed regarding the durability of negative smoking outcome expectancies. Moreover, to ensure the validity of experimental manipulations, future studies should utilize direct measures of outcome expectancies—such as participants’ beliefs about the socially undesirable consequences of smoking—rather than relying solely on behavioral intention items.

## Conclusions

The current study contributes to a deeper understanding of the underlying mechanisms that inhibit attentional bias while emphasizing the significance of external social situations and internal outcome expectancies. This work has potential implications for developing intervention strategies aimed at smoking cessation.

## Data Availability

The raw data supporting the conclusions of this article will be made available by the authors, without undue reservation.
